# Kocurin, the True Structure of PM181104, an Anti-Methicillin-Resistant *Staphylococcus aureus* (MRSA) Thiazolyl Peptide from the Marine-Derived Bacterium *Kocuria palustris*

**DOI:** 10.3390/md11020387

**Published:** 2013-02-04

**Authors:** Jesús Martín, Thiciana da S. Sousa, Gloria Crespo, Sara Palomo, Ignacio González, José R. Tormo, Mercedes de la Cruz, Matthew Anderson, Russell T. Hill, Francisca Vicente, Olga Genilloud, Fernando Reyes

**Affiliations:** 1 Fundación MEDINA, Centro de Excelencia en Investigación de Medicamentos Innovadores en Andalucía, Avda. del Conocimiento 3, Granada 18016, Spain; E-Mails: jesus.martin@medinaandalucia.es (J.M.); thicy85@yahoo.com.br (T.S.S.); gloria.crespo@medinaandalucia.es (G.C.); sara.palomo@medinaandalucia.es (S.P.); ignacio.gonzalez@medinaandalucia.es (I.G.); jose.tormo@medinaandalucia.es (J.R.T.); mercedes.delacruz@medinaandalucia.es (M.C.); francisca.vicente@medinaandalucia.es (F.V.); olga.genilloud@medinaandalucia.es (O.G.); 2 Institute of Marine and Environmental Technology, University of Maryland Center for Environmental Science, 701 E Pratt Street, Baltimore, MD 21202, USA; E-Mails: andersomenator@gmail.com (M.A.); hill@umces.edu (R.T.H.)

**Keywords:** marine-derived bacteria, *Kocuria palustris*, thiazolyl peptides, structure elucidation, antibacterial activity

## Abstract

A new thiazolyl peptide, kocurin (**1**), was isolated from culture broths of a marine-derived *Kocuria palustris*. Its structural elucidation was accomplished using a combination of spectroscopic and chemical methods, including HRMS, extensive 1D and 2D NMR analysis, MS/MS fragmentation, and chemical degradation and Marfey’s analysis of the resulting amino acid residues. The structure herein reported corrects that previously assigned to PM181104 (**3**). Kocurin displayed activity against methicillin-resistant *Staphylococcus aureus* (MRSA), with MIC values in the submicromolar range.

## 1. Introduction

The increasing resistance to known antibiotics developed by bacterial pathogens is one of the greatest threats to human health worldwide. Methicillin-resistant *Staphylococcus aureus* (MRSA) infections have received much attention and are still a major cause of death, killing more Americans every year (~19,000) than emphysema, HIV/AIDS, Parkinson’s disease, and homicide combined [1]. Therefore, there remains an urgent need to find new anti-MRSA antibiotics with novel modes of action.

Thiazolyl peptides constitute a class of naturally occurring compounds with potent *in vitro* activity against Gram-positive bacteria. The structure of these polycyclic peptides contains a central pyridine/tetrahydropyridine ring, with up to three thiazolyl substituents at the 2-, 3-, and 6-positions of the central heterocycle. This structural class includes close to 100 molecules [2], with the first member, micrococcin, isolated in 1948 [3], and some new members of the thiazomycin family reported as recently as 2009 [4]. Despite its interesting *in vitro* submicromolar antibacterial activity against MRSA, the development of this class of compounds as clinical agents has been hampered due to extremely poor physicochemical properties, most notably low aqueous solubility and unfavorable pharmacokinetics.

**Figure 1:**
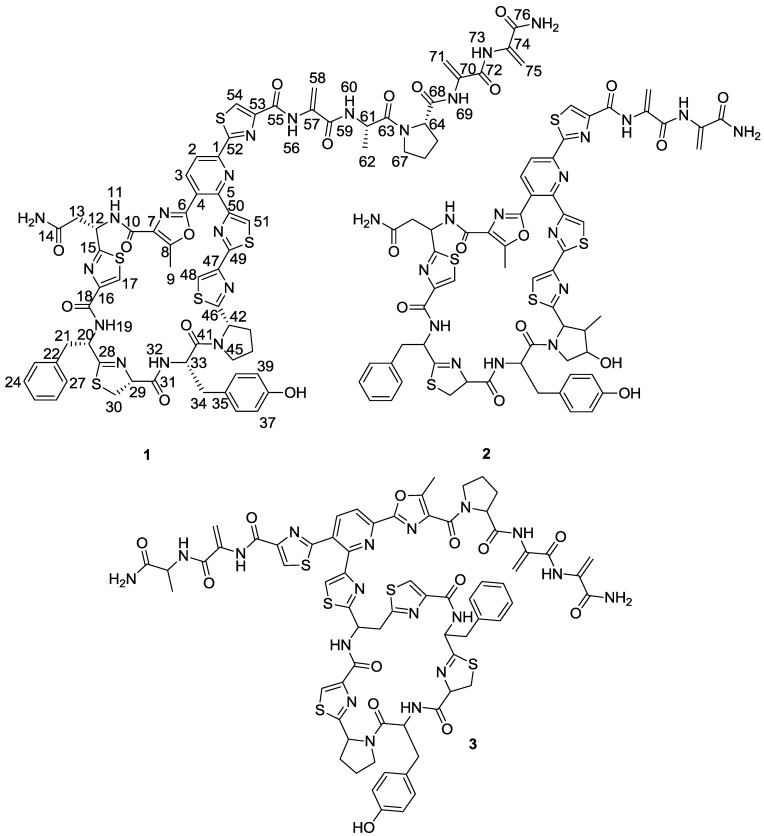
Structures of kocurin (**1**), GE 37468 A (**2**) and PM181104 (**3**).

As part of our continuing program for the search of new bioactive molecules, 44 bacterial strains of the family *Micrococcaceae* isolated from marine sponges collected in the Florida Keys (USA) were surveyed for the presence of genes encoding non ribosomal peptide synthetase (NRPS) and polyketide synthase (PKS) enzymes and their fermentation extracts were tested for antibiotic activity against clinically relevant strains: Gram-positive bacteria (*Bacillus subtilis* and methicillin-resistant *Staphylococcus aureus*), Gram-negative bacteria (*Acinetobacter baumannii*) and yeast (*Candida albicans*). Growth inhibition of MRSA was observed in acetone extracts of three of these strains [5]. This antibacterial activity was reproduced in new fermentations and the active component, kocurin (**1**) (Figure 1), was isolated from one of these strains belonging to the genus *Kocuria* by reversed phase C18 chromatography and semipreparative HPLC, and was identified as a new member of the thiazolyl peptide family of antibiotics. The structural elucidation of kocurin was accomplished using a combination of spectroscopic and chemical methods, including HRMS, extensive 1D and 2D NMR analysis, and chemical degradation and Marfey’s analysis of the resulting amino acid residues.

## 2. Results and Discussion

The producing bacterium, *K. palustris*, was fermented for 1 day in R358 medium. A 7 L fermentation was centrifuged and the cell pellet was extracted with methanol (3 × 50 mL). This extract was chromatographed on a reversed phase C18 column using a gradient H_2_O/MeOH and the bioactive fractions were finally purified by repeated semipreparative and preparative HPLC to yield 1.4 mg of **1** as a white solid.

Kocurin (**1**) was assigned a molecular formula of C_69_H_66_N_18_O_13_S_5_ by ESI-TOF mass spectrometry (*m/z* 1515.3739, calc. for [M + H]^+^ 1515.3733). Two absorption maxima in the UV spectrum at 218, 307, and a shoulder at 349 nm together with the presence of five sulphur atoms in the molecular formula of **1** identified the compound as a member of the thiazolyl peptide class. The ^1^H NMR spectrum (Table 1) displayed downfield singlets due to the presence of four thiazoles (7.45, 7.86, 8.01 and 8.09 ppm) and two doublets characteristic of a 2,3,6-trisubstituted pyridine ring (8.24 and 8.36 ppm). Other low field proton signals were attributable to protons of the aromatic rings of phenylalanine (7.17, 7.23 and 7.18 ppm) and tyrosine units (6.45 and 6.60 ppm). Three pairs of methylene groups resonating at 5.52 and 6.69 ppm, 5.48 and 6.54 ppm, and 5.38 and 6.58 ppm correlated in the HSQC spectrum with carbons at 102.8, 103.6 and 103.5, respectively, accounting for three dehydroalanine residues. Correlations observed in the HMBC spectrum from these three pairs of proton signals to carbons at 133.9 and 163.1, 134.4 and 162.0, and 132.9 and 165.3 corroborated the identity of these three structural units.

**Table 1:** ^1^H and ^13^C NMR data (500 and 125 MHz, CDCl_3_) for Kocurin (**1**).

No.	δ (C)	δ (H), Multiplicity, *J*		δ (C)	δ (C), Multiplicity, *J*
1	150.8		39	117.6	6.45, d, 8.2
2	138.6	8.36, d, 8.2	40	131.1	6.60, d, 8.2
3	118.6	8.24, d, 8.2	41	170.5	
4	122.5		42	61.7	5.33, t, 7.5
5	151.4		43	34.2	2.58, m; 2.07, m
6	155.8		44	25.7	2.37, m; 2.17, m
7	129.9		45	48.2	3.99, m, 2H
8	154.2		46	173.5	
9	12.1	2.88, s, 3H	47	148.6	
10	163.1		48	114.0	7.45, s
11		8.65, d, 9.7	49	159.7	
12	47.7	4.90, m	50	154.2	
13	37.6	2.16, m	51	122.5	8.01, s
		0.50, dd, 17.4, 3.0			
14	172.3		52	167.9	
15	171.9		53	151.4	
16	149.0		54	127.0	8.29, s
17	122.4	7.86, s	55	159.4	
18	160.3		56		10.05, s
19		8.35, d, 6.4	57	133.9	
20	53.5	5.13, m	58	102.8	6.69, br s; 5.52, br s
21	39.0	3.58, m	59	163.1	
		3.36, dd, 14.0, 3.1			
22	135.7		60		7.44, d, 7.4
23	129.9	7.23, d, 7.8	61	47.6	4.90, m
24	128.4	7.28, t, 7.8	62	18.4	1.55, d, 6.8, 3H
25	127.2	7.17, t, 7.8	63	172.3	
26	128.4	7.28, t, 7.8	64	61.1	4.71, dd, 7.8, 3.3
27	129.9	7.23, d, 7.8	65	27.8	2.38, m; 2.09, m
28	172.9		66	25.1	2.17, m; 2.09, m
29	78.3	4.83, ddd, 13.3, 8.7, 2.6	67	47.3	3.71, m, 2H
30	35.9	3.76, m; 3.58, m	68	169.6	
31	169.1		69		8.89, s
32		7.40, d, 9.3	70	134.4	
33	51.4	5.21, m	71	103.6	6.54, d, 1.9
					5.48, br s
34	36.4	3.14, dd, 14.1, 3.0	72	162.0	
		2.94, dd, 14.1, 4.5			
35	125.6		73		8.86, s
36	131.1	6.60, d, 8.2	74	132.9	
37	117.6	6.45, d, 8.2	75	103.5	6.58, d, 1.4
					5.38, br s
38	155.6		76	165.3	

Other building blocks identified in the ^1^H NMR spectrum and confirmed via COSY, HSQC and HMBC correlations were an asparagine unit (8.65, 4.90, 2.56 and 0.50 ppm), an alanine (7.44, 4.90 and 1.55 ppm), two prolines and a thiazoline ring (4.83, 3.76, and 3.58 ppm). The unusual low field chemical shift of the methine carbon of the latter residue (78.3 ppm) was due to the presence of a cyclic secondary ketimine bond in addition to the downfield effect of the adjacent carbonyl group. Finally, one methyloxazole residue was also present in the structure of **1** as revealed by the presence of a singlet methyl group at δ_H_ 2.88 ppm that correlated in the HMBC spectrum to 3 carbons at δ_C_ 129.9 (C-7), 155.8 (C-8) and 163.1 (C-10, weak, 4-bond correlation).

**Figure 2:**
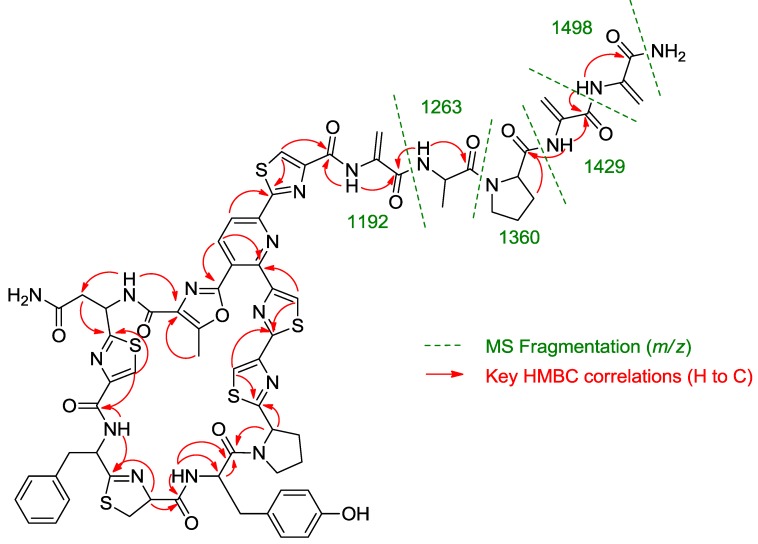
Key HMBC correlations and MS/MS fragments observed in the spectra of kocurin (**1**).

Once all the subunits present in the structure of the new peptide were identified, the sequence of amino acids was established by analysis of key correlations observed in the HMBC spectrum (Figure 2) and MS/MS analysis. HMBC correlations confirmed that the central pyridine nucleus was surrounded by two of the thiazolyl and the methyloxazole units and allowed the planar structure of the compound to be established as depicted in Figure 2. Additional support for the sequence determined by HMBC came from the analysis of key fragments observed in the MS/MS spectrum of the molecule. Thus, the sequence Dha-Dha-Pro-Ala for the *C*-terminal amino acid side chain was determined from fragments at *m/z* 1498, 1429, 1360, 1263, and 1192 (Figure 2). The planar structure of kocurin was found to be similar to that of the antibiotic peptide GE 37468 A (**2**) [6,7], the major structural differences found between both compounds being the replacement of the methylhydroxyproline unit in the cycle of **2** by proline in **1** and the presence of additional amino acid units in the side chain of the latter compound.

The absolute configurations of the amino acid residues in kocurin were determined by application of the Marfey’s methodology [8]. A hydrolysis of the sample with HCl 6 N (110 °C, overnight) and derivatization of the hydrolysate with 1-fluoro-2,4-dinitrophenyl-5-L-valinamide (L-FDVA) revealed the presence of L-Tyr, L-Pro (×1) and L-Ala on the basis of comparison of their retention times with those of derivatized standards. In order to liberate those amino acids adjacent to thiazole rings to determine their absolute configurations, it was necessary to cleave these by ozonolysis prior to hydrolysis. Treatment of a solution of **1** in CHCl_3_/MeOH with ozone for 10 min followed by acid hydrolysis with 6 N HCl (110 °C, overnight) and derivatization with L-FDVA identified L-Asp, L-Phe, L-Cya, L-Pro (×2) and L-Ala as components of the hydrolyzate by LC/MS. Tyr was not found in this analysis, probably due to its decomposition during the ozonization of the peptide. The ozonolysis of the sample prior to the acid hydrolysis had the additional advantage of reducing the epimerization of the thiazoline present in the molecule, which has been described to readily occur in the presence of mild acid or base. A 1.6:1 ratio of L- and D-Cya was obtained in the LC/MS analysis, confirming L-Cys as the residue present in the structure of kocurin. The L-Cys assignment was also supported by comparative ^1^H and ^13^C NMR data of the thiazoline in **1** to those reported for other molecules containing the same residue.

**Figure 3:**
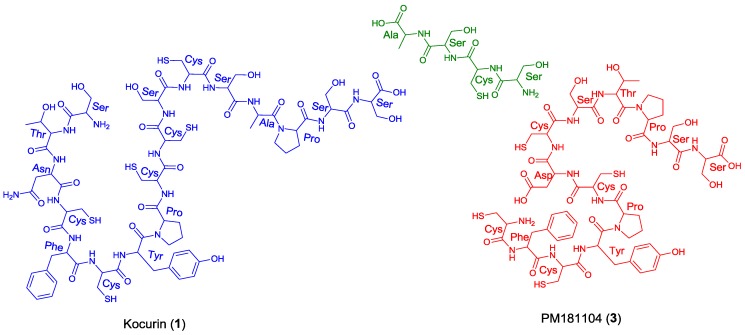
Structural units required for the biosynthesis of kocurin (**1**) and PM181104 (**3**).

Once the complete structure of the compound had been determined, a literature search revealed the existence of two patents describing compounds having the same molecular formula as kocurin, the thyazolyl peptide PM181104 (**3**), isolated from a marine-derived *Kocuria* sp. (ZMA B-1/MTCC 5269) [9] and a molecule with the same planar structure as kocurin obtained from *Kocuria* sp. Strain MI-67-EC3-038, isolated from a marine sample collected in the Southeast coast of Spain [10]. Although no explanation is given in the first patent about the rationale followed in the structural elucidation of **3**, we believe that kocurin and PM181104 are actually the same molecule. Although the same residues can be identified by NMR in both structures, on the basis of biogenetic grounds we firmly believe that the structure proposed for **3** is not correct, probably due to a misinterpretation of some HMBC correlations observed for carbons resonating very closely, and that the actual compound isolated in the work described in the patent has the structure herein proposed for kocurin. It is well known that thiazolyl peptides arise from a cascade of post-translational modifications on 50 to 60 residue pre-peptide precursors that trim away the *N*-terminal leader sequences (40 residues) while at the *C*-terminal, 14 to 18 residues are converted into the mature scaffold [11]. In this sense, kocurin would be the result of post-translational modifications of a linear C-terminal peptide containing 17 amino acids (Ser-Thr-Asn-Cys-Phe-Cys-Tyr-Pro-Cys-Cys-Ser-Cys-Ser-Ala-Pro-Ser-Ser) whereas the biosynthesis of PM181104 would require the participation of two independent peptide units of 13 (Cys-Phe-Cys-Tyr-Pro-Cys-Asp/Asn-Cys-Ser-Thr-Pro-Ser-Ser) and 4 (Ser-Cys-Ser-Ala) amino acid residues (Figure 3). A second inconsistency in the structure proposed for compound **3** is the presence of two *C*-terminal amino acids. Due to this, the MS/MS fragmentation pattern generated by this compound should contain additional ions to those observed for **1**, originated by losses of amino acids from both side chains. Among others, ions at *m/z* 1427 and 1358 accounting for the elimination of the terminal alanine and alanine-DHA residues in PM181104, not observed in the spectrum of kocurin, should appear in the MS/MS spectrum of the molecule. Unfortunately, no MS/MS fragmentation of PM181104 is reported in the patent and only the molecular ion of the compound is included as part of the structural information. Finally, a comparison of the ^1^H NMR in DMSO-*d*_6_ of kocurin with that reported in the patent for PM181104 definitely confirms that both molecules are identical.

The biological activity of kocurin was tested against a panel of bacterial and fungal pathogens, including wild-type MRSA and *S. aureus* strains resistant to thiazomycin, other Gram-positive (*Bacillus subtilis* and *Enterococcus faecium*) and Gram-negative (*Escherichia coli*, *Acinetobacter baumannii* and *Pseudomonas aeruginosa*) bacteria and one fungus (*Candida albicans*). Kocurin strongly inhibited the growth of MRSA MB5393 with a MIC value of 0.25 μg/mL. In addition, the compound also displayed antibacterial activity against *B. subtilis* and *E. faecium* in a solid agar test, with zones of inhibition (ZOI) of 9 and 10 mm when tested at a concentration of 8 μg/mL. On the other hand, the compound displayed ZOI of 5 mm when a 10 μL aliquot of a 2 μg/mL solution was spotted on plates containing cultures of both, wild-type and thiazomycin resistant *S. aureus* strains, revealing that the mechanism of action of the compound is different from that of thiazomycin. Finally, kocurin did not inhibit the growth of Gram-negative bacteria or *C. albicans* when tested at a concentration of 16 μg/mL.

## 3. Experimental Section

### 3.1. General Experimental Procedures

Optical rotations were determined using a JASCO P-2000 polarimeter. UV spectra were obtained with an Agilent 1200 DAD. NMR spectra were recorded on a Varian “INOVA 500” spectrometer at 500/125 MHz (^1^H/^13^C). Chemical shifts were reported in ppm using residual CDCl_3_ (δ 7.26 for ^1^H and 77.0 for ^13^C) as internal reference. HMBC experiments were optimized for a ^3^*J*_CH_ of 8 Hz. (+)-ESI-TOFMS was performed on a Bruker maXis spectrometer.

### 3.2. Producing Microorganism

The producing strain F-276,345 was obtained from a sponge sample collected in Florida Keys, USA [12]. Upon phylogenetic analysis based on partial 16S rRNA gene sequences of related type species, the strain F-276,345 was identified as a new member of the species *Kocuria lacustris* [5].

### 3.3. Fermentation of the Producing Microorganism

A 7.2 liter fermentation of *Kocuria palustris* F-276,345 was obtained as follows: a first seed culture of the strain F-276,345 was prepared by inoculating three 50 mL tubes containing 12 mL each of MY seed medium (D-(+)-Glucose 10 g/L, Bacto Yeast Extract 3 g/L, Proteose-peptone 5 g/L, Malt Extract 3 g/L, adjusted to pH 7.0), with 0.5 mL of a frozen inoculum stock of the producing strain and incubating the tube at 28 °C with shaking at 220 rpm for about 24 h. A second seed culture was prepared by inoculating eight 250 mL flasks containing 50 mL each of MY seed medium with 2.5 mL of the first seed. A 5% aliquot of the second seed culture was transferred to each of the forty-eight 500 mL flasks containing 150 mL of the production medium R358, which is a modified version of the one used by Jensen *et al.* [13], which consists of starch from potato 10 g/L, Bacto Yeast Extract 4 g/L, Bacto Peptone 2 g/L, FeSO_4_.7H_2_O (5 mL of a 8 g/L stock solution), and KBr (5 mL of a 20 g/L stock solution) at pH 7.0. The flasks were incubated at 28 °C for 1 day in a rotary shaker at 220 rpm and 70% humidity before harvesting.

### 3.4. Extraction and Isolation

The culture broth was centrifuged for 15 min at 8500 rpm and the supernatant was discarded. The cell pellet was extracted with MeOH (3 × 50 mL), filtered and the solvent was evaporated to dryness. The dried extract was subjected to reversed phase flash chromatography (15.5 g of RediSep C18, gradient from 20% MeOH to 100% MeOH in 12.5 min + 100% MeOH for 10 min, 10 mL/min, UV detection at 210 and 305 nm) yielding a fraction eluting between 12.5 and 14.4 min that was subjected to semipreparative HPLC (X-Bridge Phenyl, 10 × 15 mm, 5 μm, gradient H_2_O–CH_3_CN from 25% to 80% CH_3_CN in 35 min, 3.6 mL/min, UV detection) to yield 1.4 mg of kocurin (**1**) as a white amorphous solid. 

Data for kocurin (**1**): White amorphous solid; [α]^25^_D_ +27.0 (*c* 0.11, CHCl_3_/MeOH 1:1); UV (DAD) λ_max_ 218, 307, 349 (sh) nm; IR (ATR) ν_max_ 3342, 2922, 2853, 1649, 1512, 1427 cm^−1^; ^1^H and ^13^C NMR: see Table 1; (+)-ESI-TOFMS *m/z* 1515.3739 [M + H]^+^ (calc. for C_69_H_66_N_18_O_13_S_5_, 1515.3733, Δ 0.3 ppm).

### 3.5. Ozonolysis, Acid Hydrolysis, and Marfey’s Analysis of Kocurin (**1**)

A sample of kocurin (0.1 mg, 50 mM) was treated with 6 N HCl in a sealed vial at 110 °C for 24 h. The solution was concentrated to dryness *in vacuo*. The hydrolysate was reconstituted in H_2_O (50 μL) and treated with a solution of 1-fluoro-2,4-dinitrophenyl-5-L-valine-amide (L-FDVA, 150 μL, 1% in acetone) and a 1 M solution of NaHCO_3_ (20 μL) in a sealed vial at 40 °C for 1 h. The reaction mixture was neutralized with 1 N HCl (20 μL) and an aliquot (10 μL) was diluted with CH_3_CN (40 μL). The resulting solution was analyzed by LC-MS employing a Waters X-Bridge C18 column (4.6 × 150 mm, 5 μm) and a gradient elution profile of 10% B (90% CH_3_CN, 10% H_2_O, 1.3 mM TFA, 1.3 mM ammonium formiate)/90% A (10% CH_3_CN, 90% H_2_O, 1.3 mM TFA, 1.3 mM ammonium formiate) to 55% B/45% A over 35 min at flow of 1 mL/min. The hydrolysate of kocurin contained L-Ala (13.7 min), L-Pro (14.9 min) and L-Tyr (30.5 min). The retention time of the L-FDVA derivatives of the authentic amino acids were as follows: L-Ala (13.8 min), D-Ala (18.3 min), L-Pro (14.9 min), D-Pro (18.1 min), L-Tyr (30.6 min) and D-Tyr (34.8 min).

A second sample (0.2 mg) of the peptide was dissolved in 2 mL of CHCl_3_/CH_3_OH, and ozone was bubbled through the solution for 10 min. This solution was evaporated to dryness on a rotary evaporator. Subsequent hydrolysis and Marfey’s analysis of the resulting residue was performed as described above. The resulting solution was analyzed by LC-MS employing a Waters X-Bridge C18 column (4.6 × 150 mm, 5 μm) and a gradient elution profile of 10% B/90% A to 35% B/65% A over 35 min and then to 100% B in 1 min and held at 100% B for 4 min using a flow of 1 mL/min. The hydrolysate of the ozonolysis product gave L-Ala (19.0 min), L-Asp (14.1 min), L-Phe (35.2 min) and L-Pro (21.4 min). The retention time of the L-FDVA derivatives of the authentic amino acids were as follows: L-Ala (19.0 min), D-Ala (28.5 min), L-Asp (14.1 min), D-Asp (16.4 min), L-Phe (34.6 min), D-Phe (37.8 min), L-Pro (20.8 min) and D-Pro (27.3 min). A second chromatographic method using the same solvent system as the previous methods (Waters X-Bridge C18 column (4.6 × 150 mm, 5 μm) and isocratic elution 3% B/97% A over 35 min at flow of 1 mL/min) was used for the identification of the L-Cya residue (17.4 min) (Cya L:D, 1.5:1). The retention times of the L-FDVA derivatives of cysteic acid were: L-Cya (17.4 min), D-Cya (18.5 min).

### 3.6. Antifungal Assays

Frozen stocks of *C. albicans* were used to inoculate Sabouraud Dextrose Agar (SDA) plates for confluent growth. Plates were incubated for 24 h, at 35 °C. The grown colonies were harvested from the SDA plates and suspended in RPMI-1640 modified medium. Modified RPMI-1640 medium was prepared as follows: 20.8 g of RPMI powder (Sigma) were poured into a 2 L flask, together with 13.4 g of YNB, 1.8 L of milliQ water, 80 mL of 1 M HEPES and 72 mL of glucose 50%. The volume was adjusted to 2 L and filtered. The OD_660_ was adjusted to 0.25 using RPMI-1640 modified as diluent and blank. This inoculum was diluted 1:10 and kept on ice until used to inoculate 96-well microtiter plates. For the assay, 90 μL/well of the 1:10 diluted inoculum were mixed with 1.6 μL/well of compound solution in DMSO and 8.4 μL/well of RPMI-1640 modified medium. Amphotericin B and Penicillin G were used as internal positive and negative controls respectively. After dispensing the inocula, the samples and the controls, the assay plates were read in a Tecan Ultraevolution spectrophotometer at 612 nm for *T*_0_ (zero time). Then, the plates were statically incubated at 37 °C for 20 h. After incubation, the plates were shaken in a DPC Micromix-5 and read again for *T_f_* (final time). Percentage of growth inhibition was calculated using the following equation:

% Inhibition = 100 × {1 − [(*T*_f Sample_ − *T*_0 Sample_) − (*T*_f Blank_ − *T*_0 Blank_)]/[(*T*_f Growth_ − *T*_0 Growth_) − (*T*_f Blank_ − T_0 Blank_)]} (1)

Compound **1** was serially diluted in DMSO with a dilution factor of 2 to provide 10 concentrations starting at 160 μg/mL. The MIC was defined as the lowest concentration of an antimicrobial or antifungal compound that inhibited ≥95% of the growth of a microorganism after overnight incubation. The data were analysed using the Genedata Screener program (Genedata AG, Switzerland). In all experiments performed in this work the RZ’ factor obtained was between 0.85 and 0.95.

### 3.7. Antibacterial Assays

For the liquid media antibacterial tests, thawed stock inocula suspensions from cryovials of each microorganism (MRSA, *A. baumannii*, *E. coli* and *P. aeruginosa*) were streaked onto Luria-Bertani agar plates (LBA, 40 g/L) and incubated at 37 °C overnight to obtain isolated colonies. Single colonies of each microorganism were inoculated into 10 mL of Luria-Bertani broth medium (LB, 25 g/L in 250 mL Erlenmeyer flasks) and incubated overnight at 37 °C with shaking at 220 rpm and then diluted in order to obtain assay inocula of approximately 1.1 × 10^6^ CFU/mL (MRSA) or 5–6 × 10^5^ CFU/mL (*A. baumannii*, *E. coli* and *P. aeruginosa*).

For the assay 90 μL/well of the diluted inoculum were mixed with 1.6 μL/well of each compound dissolved in DMSO and 8.4 μL/well of LB medium. Kanamycin and amphotericin B (MRSA), rifampicin and amphotericin B (*A. baumannii*), novobiocin and amphotericin B (*E. coli*), and ciprofloxacin and amphotericin B (*P. aeruginosa*) were included as internal plate controls. Absorbance at 612 nm was measured with a Tecan UltraEvolution spectrophotometer (Tecan, Durham, USA) at *T*_0_ (zero time) and immediately after that, plates were statically incubated at 37 °C for 20 h. After this period, the assay plates were shaken using the DPC Micromix-5 and once more the absorbance at OD 612 nm was measured at *T_f_* (final time). Percentage inhibition of growth was calculated using the same equation previously described for *C. albicans*.

Assays in solid agar plates were performed in order to determine the antimicrobial susceptibility of kocurin against *B. subtilis* MB964, *E. faecium* MB5571 resistant to vancomycin and β-lactam antibiotics and thiazomycin-resistant *S. aureus* strains. The *B. subtilis* MB964 assay plates were prepared by adding 1 mL (1.5 × 10^8^ CFU/mL) of a spore suspension (Difco) directly to 1 liter of cooled nutrient agar yeast extract medium (NAYE, nutrient agar 23 g/L, yeast extract 2 g/L). The *E. faecium* strain was grown in a shaken (220 rpm) culture of brain heart infusion broth (BHI, 37 g/L) at 37 °C. The assay plates were prepared by inoculating the same cooled media plus agar (15 g/L) with 3.3% of the inoculum adjusted to an optical density (OD) range of 0.22–0.35 at 660 nm. The wild-type *S. aureus* Smith MB2865 and thiazomycin-resistant *S. aureus* MB5832 strains were grown in 10 mL of LB medium overnight. Overnight growth was adjusted to an optical density of 0.2 at 600 nm. The suspension was then added to molten LB agar in the proportion of 25 mL/L. The activity was determined by measuring the differences of the zone of clearances observed between the wild type versus the resistant plate. Compound **1** was serially diluted in DMSO 20% with a dilution factor of 2 to provide 10 compound concentrations starting at 128 μg/mL for all the agar assays. Ten microliters of each compound concentration were applied to the agar plate containing the pathogenic microorganism.

## 4. Conclusions

A thiazolyl pepetide bioactive against MRSA, kocurin (**1**), has been isolated from cultures of the marine sponge-derived bacterium *Kocuria palustris*. Its challenging structural elucidation was completed using a combination of spectroscopic and chemical methods, including HRMS, extensive 1D and 2D NMR analysis, MS/MS fragmentation, and chemical degradation and Marfey’s analysis of the resulting amino acid residues. The structure herein proposed for the compound corrects that previously reported for PM181104 in a US patent. The identification of a new bioactive compound in a member of the *Microccocaceae* suggests that this family of actinomycetes represents an alternative source for the discovery of new medically relevant small molecules. The relative abundance in marine environments, intra-familiar diversity, and ease of growth of these bacteria makes them an attractive target for the discovery of new antibiotics.

## Supplementary Files

Supplementary File 1 Supplementary Information (PDF, 189 KB)
