# Supplementary material for: Kocurin, the True Structure of PM181104, an Anti-Methicillin-Resistant Staphylococcus aureus (MRSA) Thiazolyl Peptide from the Marine-Derived Bacterium Kocuria palustris
Source: Mar Drugs. 2013 Feb 4;11(2):387–98. doi: 10.3390/md11020387 (PMC3640387; doi:10.3390/md11020387)

Supplementary Information

Figure S1. ^{1}H NMR Spectrum ($CDCl_3$, 500 MHz) of Kocurin (**1**).
Figure S2. ^{13}C NMR Spectrum ($CDCl_3$, 500 MHz) of Kocurin (**1**).
Figure S3. COSY Spectrum ($CDCl_3$, 500 MHz) of Kocurin (**1**).
Figure S4. HSQC Spectrum ($CDCl_3$, 500 MHz) of Kocurin (**1**).
Figure S5. HMBC Spectrum ($CDCl_3$, 500 MHz) of Kocurin (**1**).
Figure S6. ^{1}H NMR Spectrum (DMSO-d_6, 500 MHz) of Kocurin (**1**).
Figure S7. ESI-TOF Spectrum of Kocurin (**1**).
Figure S8. MS/MS Spectrum of Kocurin (**1**).
Figure S9. UV Spectrum of Kocurin (**1**).

Figure S1. ^{1}H NMR Spectrum ($CDCl_3$, 500 MHz) of Kocurin (**1**).

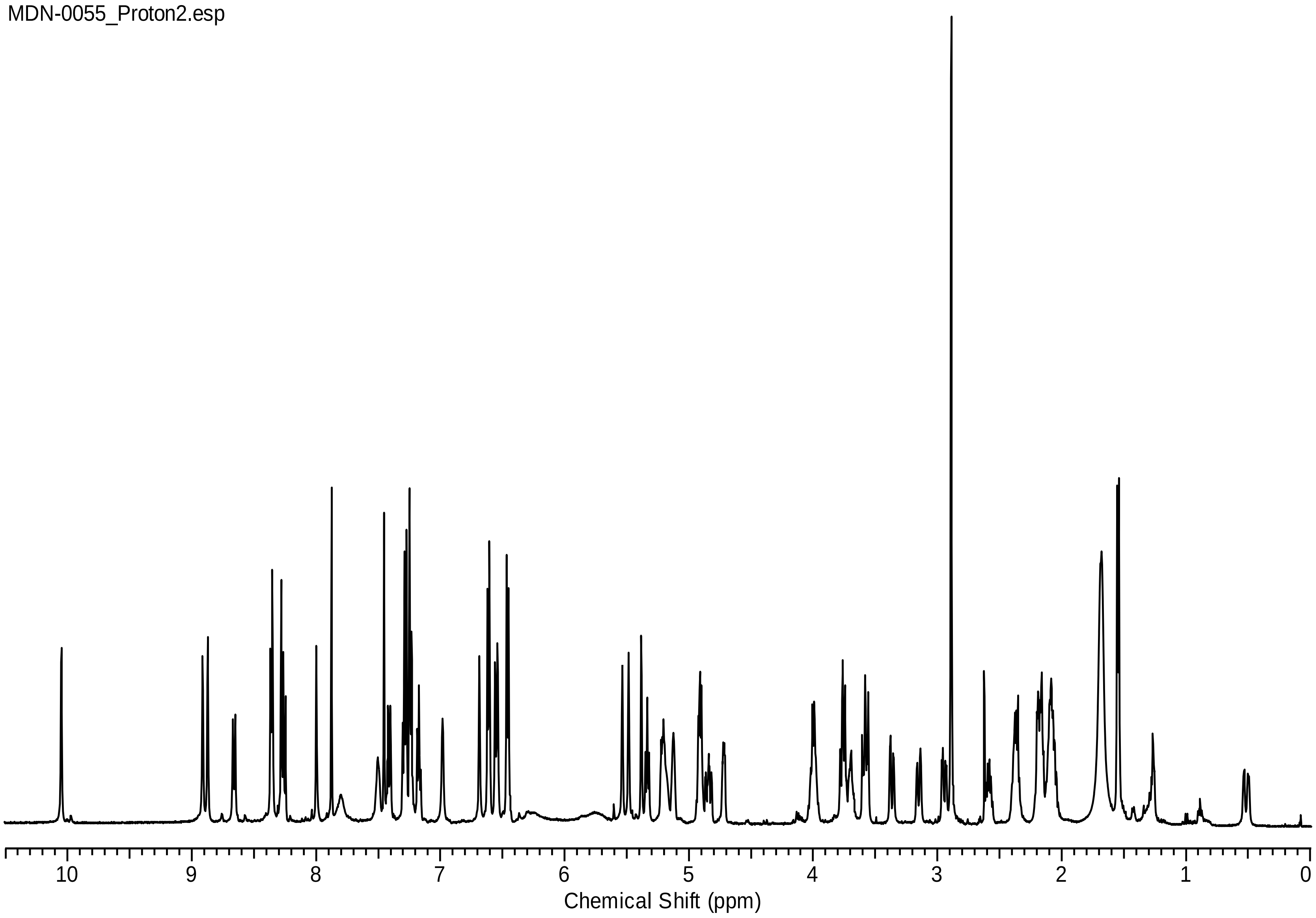

Figure S2. ^{13}C NMR Spectrum ($CDCl_3$, 500 MHz) of Kocurin (**1**).

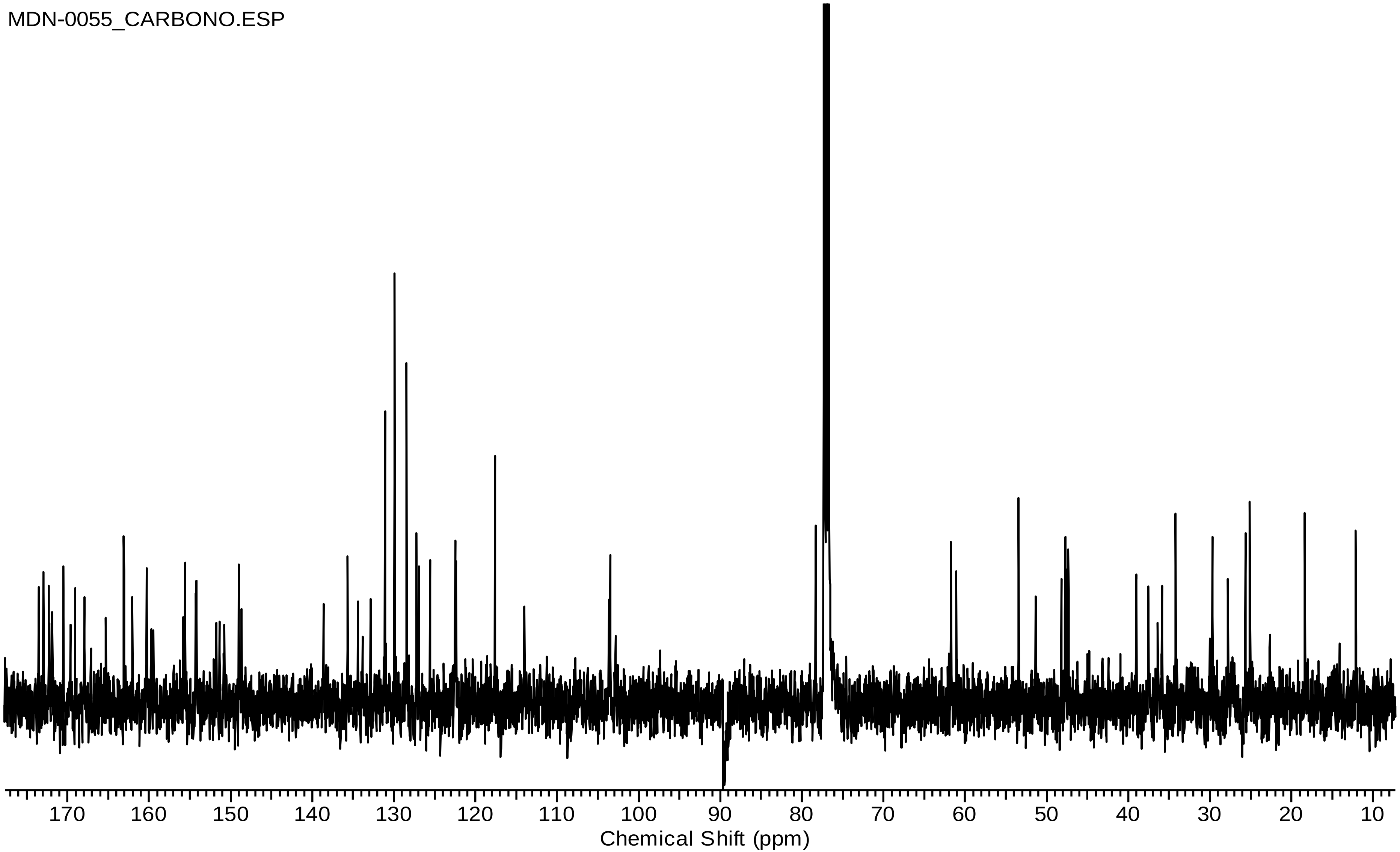

Figure S3. COSY Spectrum ($CDCl_3$, 500 MHz) of Kocurin (**1**).

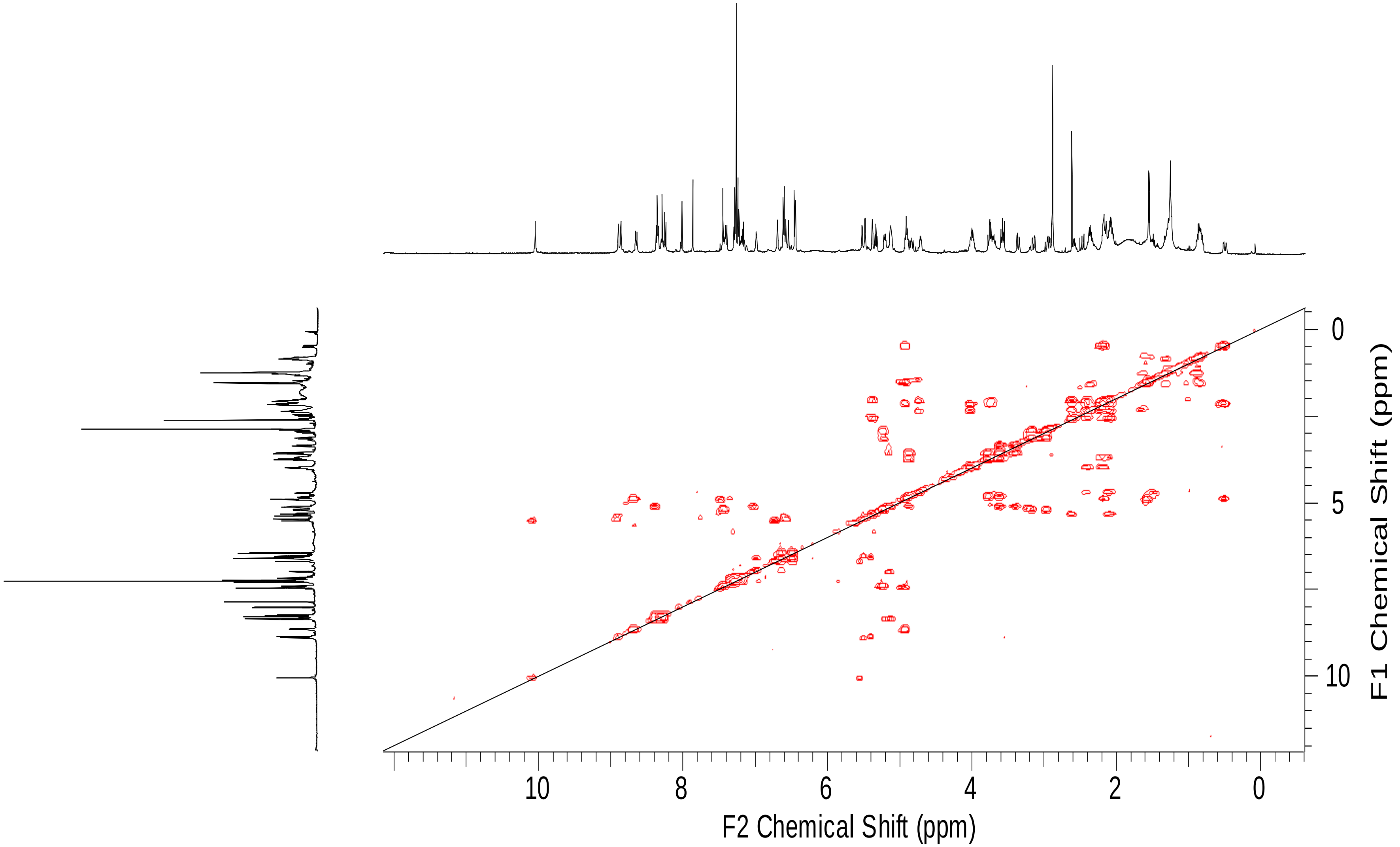

Figure S4. HSQC Spectrum ($CDCl_3$, 500 MHz) of Kocurin (**1**).

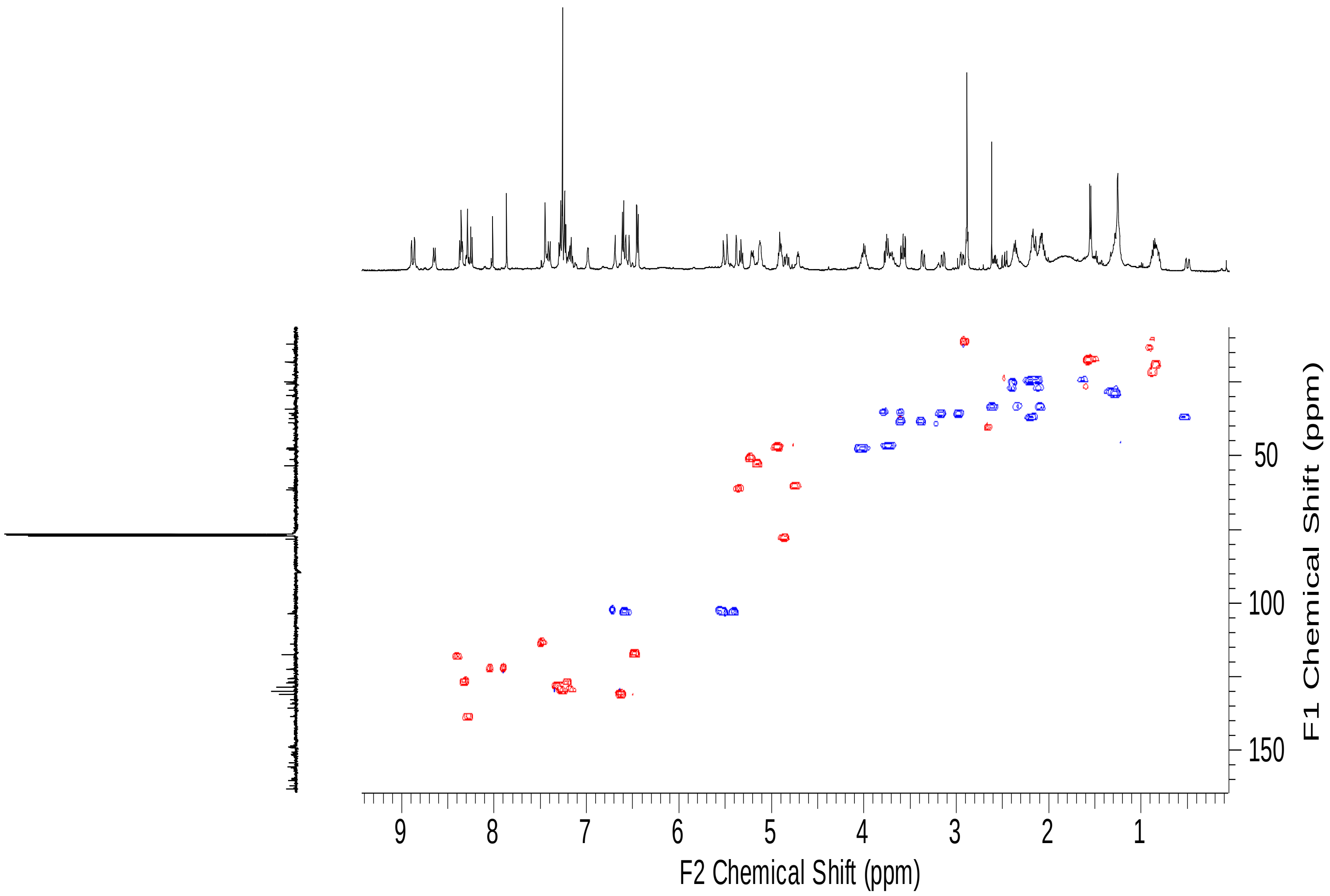

Figure S5. HMBC Spectrum ($CDCl_3$, 500 MHz) of Kocurin (**1**).

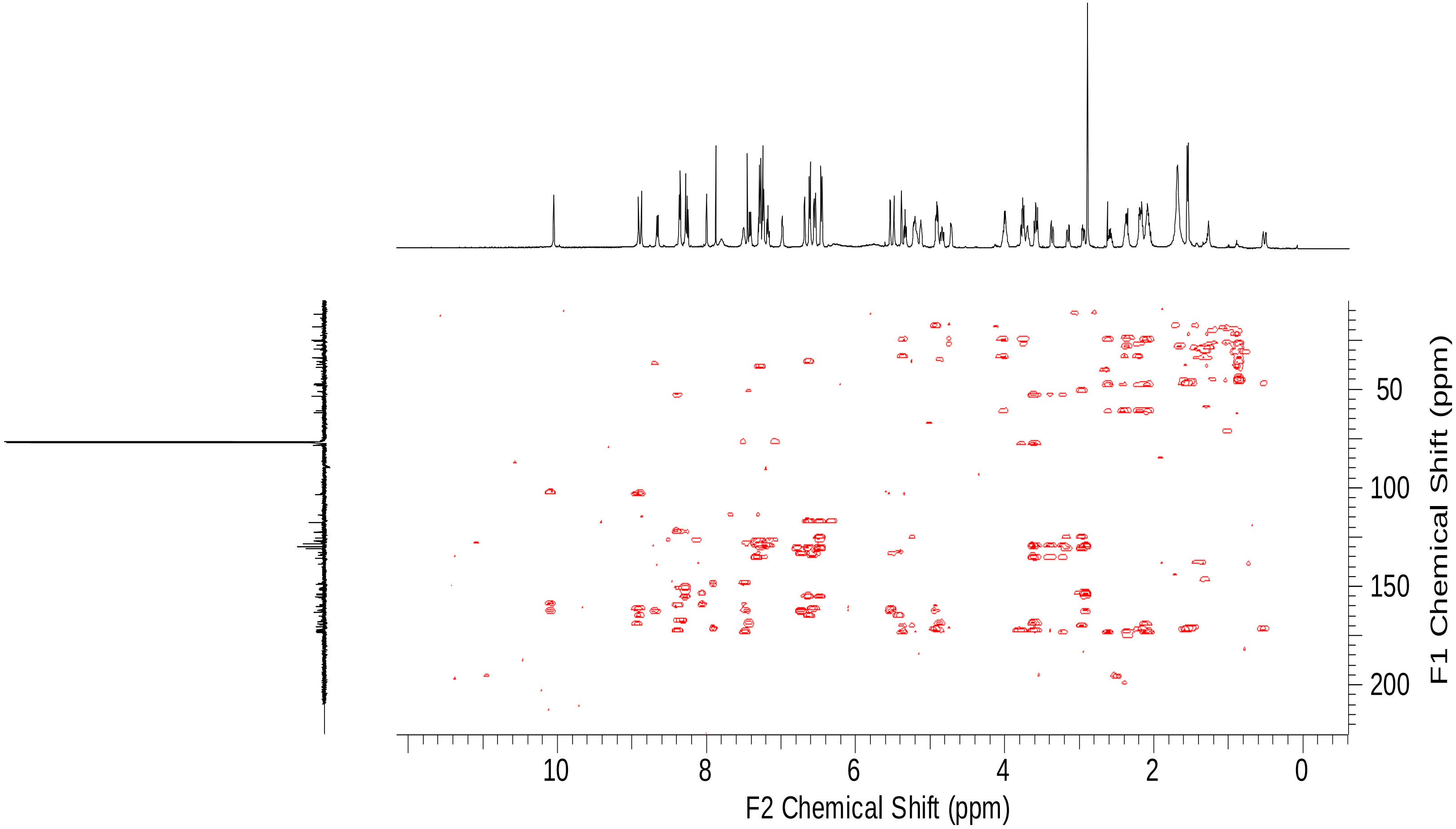

Figure S6. ^{1}H NMR Spectrum (DMSO-d_6, 500 MHz) of Kocurin (**1**).

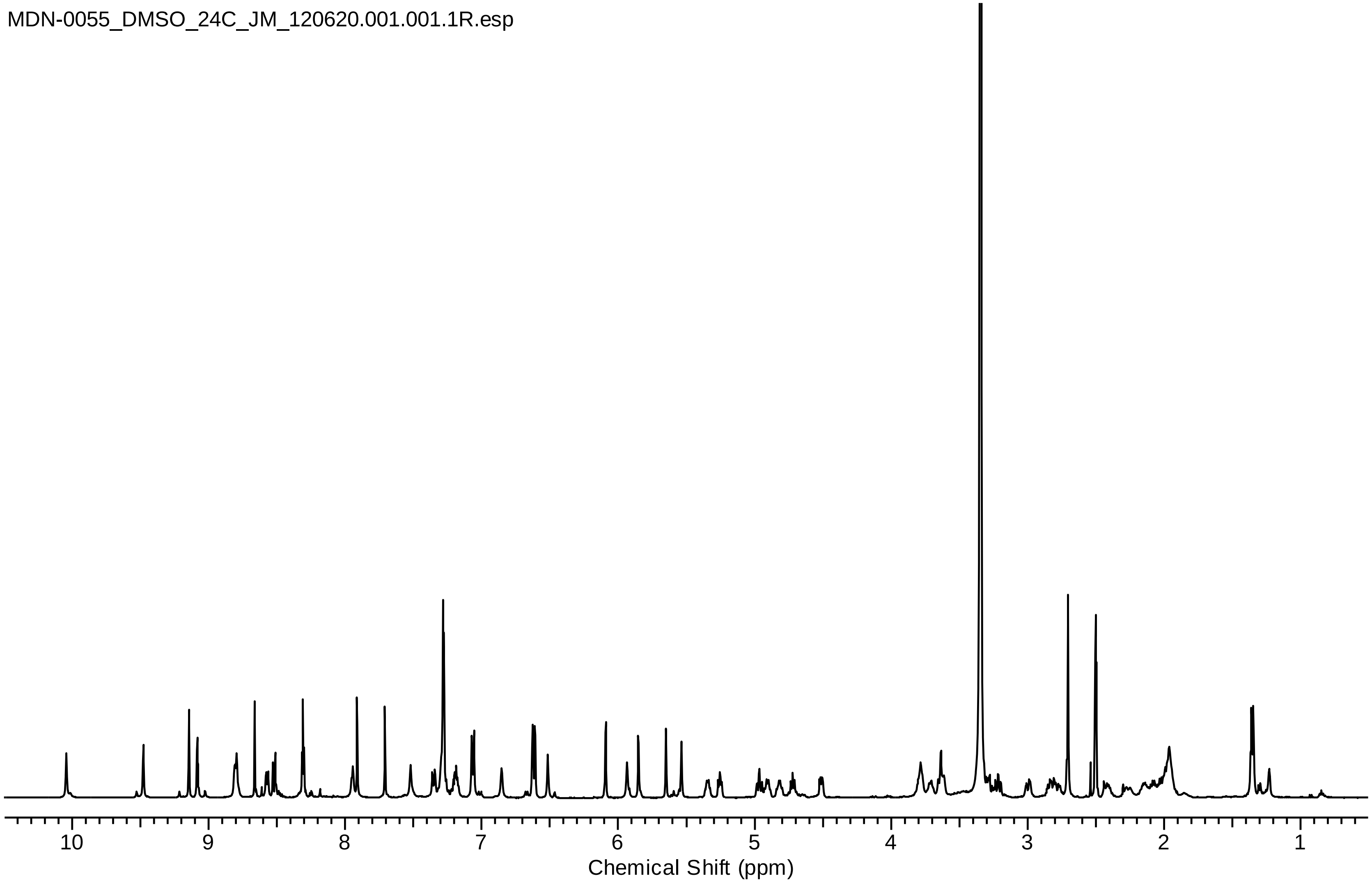

Figure S7. ESI-TOF Spectrum of Kocurin (**1**).

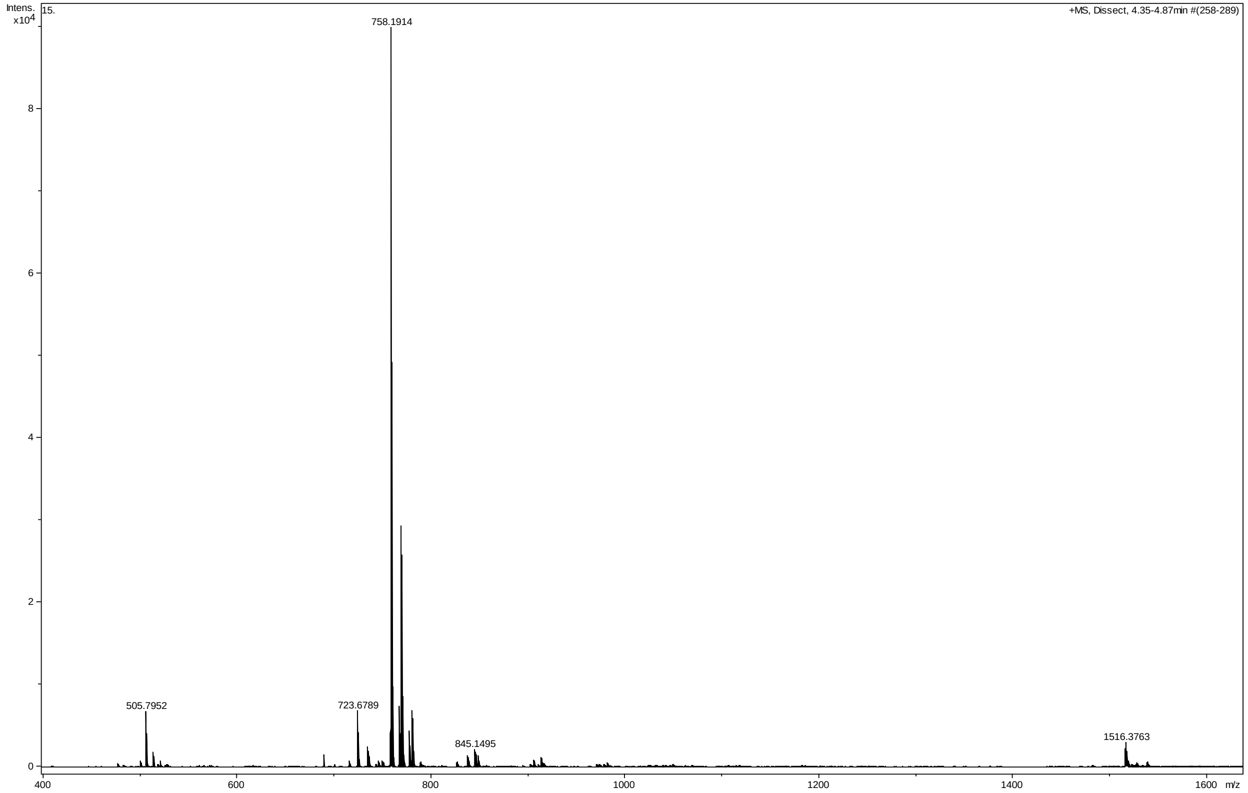

Figure S8. MS/MS Spectrum of Kocurin (**1**).

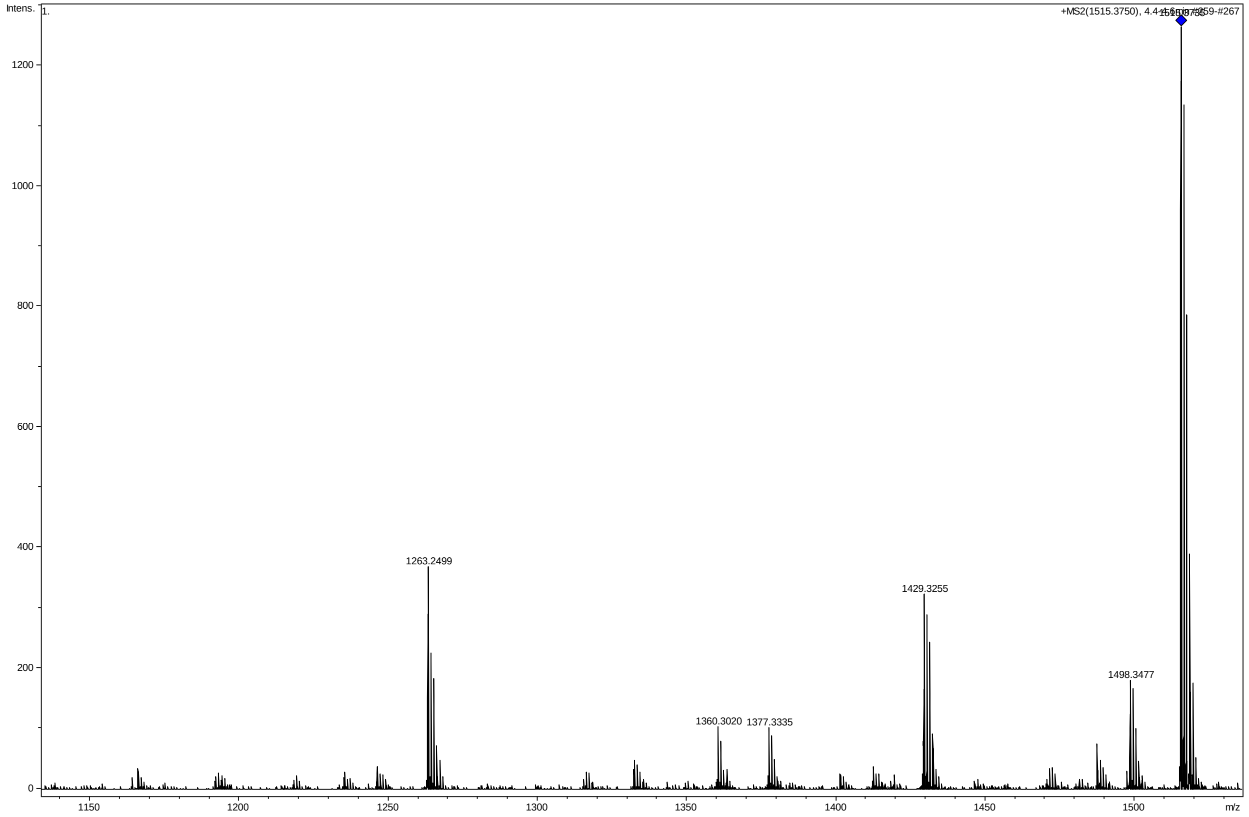

Figure S9. UV Spectrum of Kocurin (**1**).

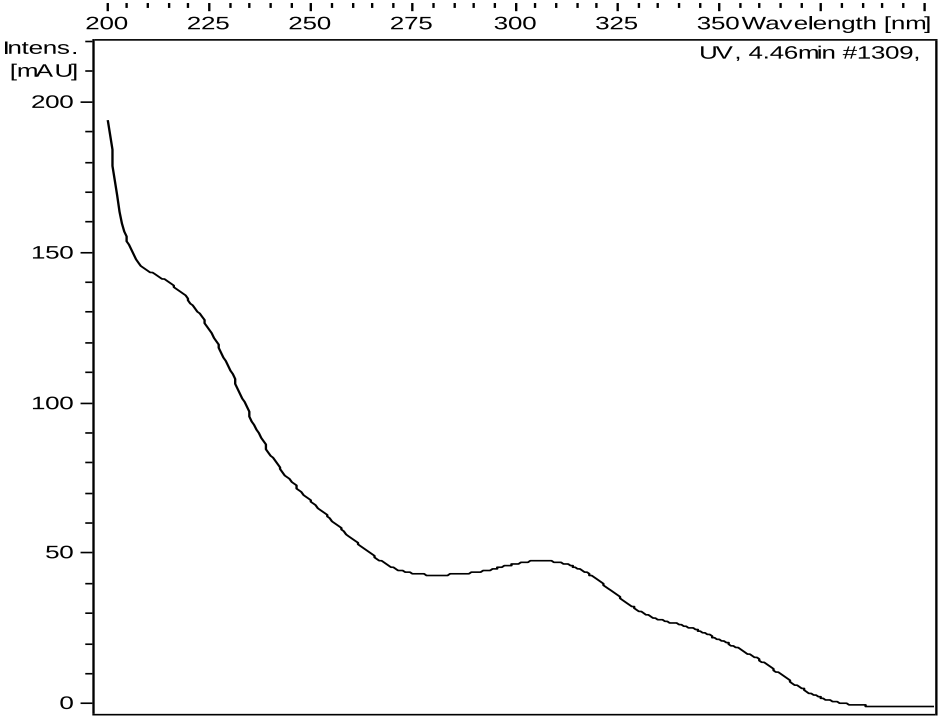

Supplement: Supplementary File 1 — Supplementary Information (PDF, 189 KB) [file marinedrugs-11-00387-s001.pdf]
